# An Enquiry into the Pathology, Causes, and Treatment of Puerperal Fever: Being an Essay for Which the Fothergillian Gold Medal Was Conferred on the Author, by the Medical Society of London, in March, 1835

**Published:** 1836-10

**Authors:** 


Art. II.—*An Enquiry into the Pathology, Causes, and Treatment of Puerperal Fever: being an Essay for which the Fothergillian Gold Medal was conferred on the Author, by the Medical Society of London, in March,* 1835. By G. Moore, f.r.c.s. *&c.—London,* 1836. 8vo. pp. 247.

We cannot look upon this work merely as a digest of literature on the subject of puerperal fever: it claims a much higher rank; for Mr. Moore has canvassed the merits of the various opinions entertained by authors upon it, and corroborated here and there the various facts from the results of his own experience.

One of the great faults of the many authors upon puerperal fever is the having described a variety of affections under the same name; an oversight which has given rise to great confusion and contrariety of opinion. Nothing is more important than to set out with distinct notions of the precise form of the disease in question, but unfortunately, in the present case, the having appeared in the puerperal state, and partaking more or less of a febrile or inflammatory character, has too often been deemed a sufficient reason for bringing under the head of puerperal fever affections differing exceedingly in their nature and treatment. This has been ably set forth by the author under the head of Preliminary Remarks, which are well worthy the attention of the obstetric practitioner.

Having taken a condensed or summary view of the symptoms of this disease as they are given by different authors, he comes to the following deduction:

"On a close inspection of this condensed and accumulated evidence, we shall discover that the summary of symptoms belonging to this formidable disease contains all the essentials of synochus: debility of corporeal and mental faculties, increased animal heat, disordered secreting functions, accelerated circulation, and excessive thirst. These constitute a perfect definition of fever according to the best authorities, and the only peculiarity in this affection appears to be the superaddition of abdominal pain. The action of the heart and arteries is greatly influenced from the earliest period of the disease; and that this is not the effect of inflammation appears from the circumstance that no other indication of disorder can be detected at the commencement of the attack. This great frequency of the pulse without any apparent reason has frequently led to the discovery of the disease, sometime before the patient has complained." (P. 34.)

The conclusion which the author thus arrives at is very important, and tends to confirm the opinion which we have long entertained,—viz. that that particular form of childbed affection which we are disposed to regard as especially, if not exclusively, entitled to the name of *the real and genuine puerperal fever*, strictly belongs to that class of diseases the fundamental character of which is a morbid condition of the blood, produced by the introduction of some deleterious agent into the circulation. It is to the last of the three species of puerperal affections so well distinguished by Dr. Douglas, of Dublin, that we wish, on the present occasion, to restrict the name of Puerperal Fever. It is "the really contagious or epidemical fever."

"The sensorium (says Dr. Douglas, quoted by the author,) is seldom in any degree disturbed: whereas in the others it is so frequently, and even sometimes is excited to high delirium. The pulse here is usually, from the moment of the attack, soft, weak, and yielding, and in quickness often exceeds 150; whereas in the first species it is full, bounding, and often incompressible; and in the second small, hard, and contracted, and in both moderately quick. The eye, instead of being suffused with a reddish or yellow tint, as in the others, is here generally pellucid, with a dilated pupil. The countenance, instead of being flushed as in the others, is here pale and shrunk, with an indescribable expression of anxiety; an expression altogether so peculiar, that the disease could on many occasions be pronounced or inferred from the countenance alone. The surface of the body, instead of being, as in others, dry and of pyrexial high heat, is here usually soft and clammy, and of heat not above the natural temperature; and not only is the skin cool with clammy exudation, but the muscles, to the impression of the finger, feel soft and flaccid, as if deprived of their vis vitæ by the influence of contagion. Indeed, there is such prostration of muscular strength and depression of vital principle from the very outset of the attack, that I must suppose the contagion to act on the human frame through the medium of the nervous system in a manner analogous to that of the contagion of the plague." (P. 42.)

The author's diagnosis of this disease is simple and practical: we could have wished that he had not attempted to distinguish hysteritis from uterine phlebitis; two diseases very near akin to each other in every respect, even in a practical point of view. He, with much judgment, points out the errors of several modern authors on this subject.

In speaking of "the usual appearances on dissection," he notices a fact which has not been so duly considered by morbid anatomists as it deserves. "When the patient is rapidly destroyed by the violence of the disease, the morbid changes bear no proportion to the severity of previous symptoms: a dubious trace of inflammation, a little bloody serum, or a few feeble adhesions, are all that dissection under such circumstances displays." (P. 63.) Among the "appearances on dissection," we certainly expected to see the appearances and condition of the blood noticed; but, beyond the single observation, that "the blood is commonly darker and more fluid than usual," (p. 65,) he does not favour us with a single remark upon this important point, until at a later part of the work; and he here devotes several pages to investigate the state of the blood in puerperal fever. Having given the observations of Gordon, Hey, and Campbell on the appearances of the blood when drawn, as tending to show its inflammatory condition in this disease, (observations which, we think, should be received with great caution, from the uncertainty of the precise form of the epidemics which these authors have described,) Mr. Moore remarks:

"In the latter stages of the disease, the blood frequently presents the same phænomena as in other adynamic and malignant fevers. From the indisposition to coagulate, both during life and after death, being always greater in proportion to the adynamia, it has been argued that the vitality of the blood is diminished in the latter stages and worst forms of this disease. The rapid circulation, which is so invariable a symptom of malignant puerperal fever, is alone sufficient to account for certain changes in the blood, since it has been satisfactorily shown that such excitement and acceleration of the heart's action speedily exhaust the vital power. Deficiency of nervous influence must tend to deteriorate this fluid, since it is proved to be so manifestly dependent on the proper activity of the nervous system for its distribution and vitality." (P. 185.)

In making these observations, our author has, in our opinion, mistaken cause for effect. We believe that it is *not* the rapid circulation which produces the changes in the blood, but a morbid condition of this fluid, in which, its vitality being more or less destroyed, it is no longer capable of maintaining the regular action of the heart, but impels it to that unwonted rapidity which characterizes the pulse in this disease. We believe that it is *not* the deficiency of nervous influence which primarily tends to deteriorate this fluid, (although it may possibly react in this way afterwards,) but the deteriorated condition of this fluid which renders it incapable of supplying the brain and nervous system with their due degree of energy. It is owing to the "vitiated and vapid state of the blood," as Dr. Stevens has shown in so masterly a manner, when speaking of contagious fevers, that frequently "there is not one symptom of inflammation during the fatal progress of disease, nor one inflammatory spot to be seen after death to mark its existence, or to induce us to believe that anything but functional disease had existed in any of the solids; yet these are the very cases, of all others, which are most fatal."

In an article in our second Number, (page 559,) we stated our belief that the physiological researches, especially during the last thirty years, both in this country and the continent, had satisfactorily proved that most, if not all, of the agents which exert such destructive energies on the nervous system do it through the medium of the circulation; as shown by the experiments of Christison and Coindet, of Brodie, Emmert, Viborg, and others. And we are much mistaken if future researches do not prove this equally of what we term the true puerperal fever. We, therefore, regret much to find no remark by the author on the state of the blood in the vessels after death. The entire absence of coagulation, the perfect fluidity, approaching both in colour and consistence to thin watery claret, have been scarcely noticed: the facts, it is true, have been hinted at, but not so distinctly brought forward as their importance demands, and, we think, not in their proper place. The black precipitate which the author observed in the blood of a person labouring under the adynamic form of this disease, tends to confirm the above views.

With such facts before them, morbid anatomists are not, we apprehend, justified in attributing this disease to a peculiar inflammatory affection of the peritoneum and abdominal viscera.

"The most legitimate conclusion to be deduced from the fact that these varieties" (described by Dr. Lee,) "arise under the same circumstances, would appear to be, that the specific and essential malady denominated puerperal fever depends not for its origin or maintenance on the inflammation which, in its progress, may exert a disorganizing influence on those structures to which it happens to be directed. The fever and the inflammation probably result from a common cause, which acts directly on the vital functions associated with those organs affected, either by an impression produced immediately through the nervous system, by the propagation of morbid matter from without, or by the circulation of deteriorated blood." (P. 83.)

It is well known to most of our readers that Dr. Lee, who has devoted much time and attention to this subject, and whose authority in obstetrical pathology is justly so high, is opposed to the view which the author advocates. Dr. Lee considers that the various morbid changes, "decidedly the effect of inflammation," account "in a most satisfactory manner for the constitutional disturbance observed during life." Mr. Moore's remarks on these views are well worthy of perusal.

"If indeed the destructive febrile affections which follow parturition, in all the various forms they assume,—inflammatory, congestive, typhoid,—depend on whether the serous, muscular, or venous tissue of the uterus be affected, it will of course follow, as a general inference, that uterine inflammation is essentially their cause. But this assumption, which is advanced as the result of extensive observation by the author above quoted, is merely begging the question at issue, and signifies only that febrile affections accompany the various forms of uterine inflammation. That these forms of inflammation are the proximate causes of the various febrile affections, is most completely refuted by the detail of his own experience, as relates to the varieties occurring under similar circumstances." . . . "It is evident that the disordered action must precede the change of structure, since we find that puerperal disease, exhibiting all the characteristic symptoms,—a rapid pulse, with pain of abdomen and pyrexia, frequently occurs, which rapidly proves fatal, and not a vestige of inflammation is to be detected in any of those tissues. Yet doubtless, were the disease to continue some time before it proved fatal, the evidence of its action might always be detected in the tissues; for continued disorder invariably produces organic alterations." (P. 89, 90.)

In following the author's quotations and observations respecting the causes and concomitants of puerperal fever, we can scarcely agree with Dr. Hamilton in considering the peculiarly fœtid discharge from the uterus as quite characteristic of the disease, because numerous cases of the worst sort have come before us, where no peculiar change in the quality of the lochia were perceived; the lochia were sparing, and ceased again in a few hours, or did not appear at all. In many cases, however, we have had ample grounds for concurring with Dr. Hamilton in attributing the virulence of the affection to "the imbibition or absorption of this self-generated poison."

The only question in our mind is, whether a disease thus produced be genuine puerperal fever, or is not rather uterine phlebitis, with or without peritonitis, accompanied by typhoid symptoms. Dr. Copland appears to support a similar opinion. It would be difficult to draw the precise line of demarcation between the two affections: their extremes are, it is true, far enough asunder; but where uterine phlebitis is accompanied, as is frequently the case, with low ataxic fever, (both probably arising from the same source, viz. the introduction of poisonous matter,) it is hardly possible to distinguish them. Well has the author observed, (p. 126,) that we must "look for somewhat beyond the production of simple phlebitis, in order to account for any kind of puerperal fever." This applies equally to peritonitis, hysteritis, &c. "In puerperal fever, as in typhus, cholera, and other epidemic or contagious diseases, which seem properly to belong to the class Neuroses, there is, besides that of inflammatory action, another element, unknown, but which has an essential influence upon the intercurrent phlegmasiæ arising in their course, and which may yield at one point only to appear at another." (P. 126.)

"A peculiar virus (says the author in the following pages,) seems to prevail throughout the whole system, and operates most speedily on those parts most excited or most debilitated. This virus seems of a specific nature, and may be capable of producing contagion. Its probable influence is well exemplified in an experiment conducted by Mr. Leuret: he injected some blood from a living horse infected with gangrenous boils *(pustule maligne)* directly into the veins of a mare five months with foal. She died in five days afterwards. The heart, lungs, and intestinal canal were studded with dark ecchymosis; the uterus was gangrenous; and the blood was dissolved and dark coloured." (P. 127-8.)

For further illustration of this subject, we refer our readers to the observations we have already made thereon in our second Number, (p. 559.)

In the succeeding pages the author appears to lose sight of the great point for which he has been so ably contending: he seems for a time to be carried away by the late prevailing opinion of puerperal fever being produced by the formation of pus in the veins, the result of uterine phlebitis, and strains at the conclusion "that a peculiar condition of the system, induced by some virus acting upon the nerves, predisposes to uterine phlebitis, and renders it malignant." That pure healthy pus does not exert a poisonous action upon the blood is now pretty generally acknowledged; and we agree with the author in even doubting whether the pus produced be ever carried into the circulation, as the vein, from swelling of its parietes, deposits of fibrine, &c., becomes more or less obliterated. It should be remembered that the injury to the circulating current had been produced even before the inflammation of the vein was established, and therefore long anterior to the formation of pus.

Although the author quotes an observation of M. Dupley, that he has never found abscess in the joints, &c., we cannot help feeling surprised that he has made no mention (as far as we can perceive,) of those peculiar effects which are occasionally observed during attacks of genuine malignant puerperal fever. Dr. Collins's observations on this head (which we have also quoted in our third Number, p. 95,) are well deserving of attention. The slight degree of pain, in no proportion to the sudden and rapid sinking of the vital powers; the absence of inflammation upon dissection; the appearance of diffuse cellular inflammation in the extremities, the decomposed condition of the muscles in these parts, and the perfect fluidity of the blood, are facts which ought not to have been passed over.

Able and satisfactory as we freely acknowledge the author's arguments to be against the assertions that the phænomena of puerperal fever are the result of inflammation, nevertheless, the enumeration of such facts as those to which we have just referred would have been of themselves a sufficient proof of the correctness of Mr. Moore's views. Our own observations, which follow the above-mentioned passage in our review of Dr. Collins's work, we also recommend to the reader's notice.

The only allusion which we can find to the peculiar deposits of matter just referred to, is at p. 139, where he says, "the existence of phlebitis, or purulent formation, either in the veins or loose tissues, may be only an accidental coincidence with this as with other fevers;" a conclusion which, after the facts the author himself has brought forward, we did not expect; and more especially because, a little further on, we meet with observations which, as far as we at present know of the subject, satisfactorily explain the formation of these remarkable deposits.

"As when the liver or the kidney is incapable of performing its proper function, deposits of bile, or of urinous fluid occur in various parts of the body, may not pus, in like manner, become a vicarious production, when the system fails to be relieved of its superabundant fibrine by the common outlets of excretion"? (P. 142.)

Whether nature endeavours to rid herself by this means of a superabundance of fibrine which is not removed by the common outlets of excretion, or whether it be an effort to relieve the circulating system of the morbid principle with which it has been contaminated, we will leave our readers to conclude from what has been already stated.

That purulent deposits are frequently found in the veins and absorbents in fatal cases of puerperal fever, is allowed on all hands; but we cannot concede that these phænomena are the characteristics of puerperal fever: they are the results of a concomitant disease, viz. inflammation of the uterine veins and absorbents, a disease which as frequently arises from the same cause. The author also mentions the remarkable circumstance of inflammation and destruction of the eye occurring in these cases: we can however, from our own experience, assure him that it is *not always* the left eye which is affected.

The observations which he has made under the head of "Contemporary Diseases" are very interesting, and tend to strengthen our view respecting the generic character of puerperal fever.

"Dr. Gordon, of Aberdeen, remarks that 'with it, and at the same time, epidemic erysipelas began, progressed with equal pace, arrived at its acme, and terminated together.' He also says, that a very frequent crisis of the disease was an external erysipelas. Mr. Hey remarks, that infectious fevers were common at the time, and he does not recollect ever having seen such malignant cases of erysipelas as then. Dr. Clarke also observes, that those inflammatory diseases which occurred were principally erysipelatous. Dr. Armstrong states that, in 1813, (the year of its greatest prevalence throughout England,) low fever, typhus, and acute rheumatism, also prevailed to an uncommon degree." (P. 164.)

These facts have been confirmed by many other observers. That "erysipelas is a contagious and infectious disease, and consequently that it is caused by the agency of a morbid poison," is an opinion recently supported by Dr. Williams,* by facts and arguments which claim attentive consideration. That phlebitis is a disease in which "speedily the symptoms assume the character of typhus," and where "there can be no question that the symptoms do very much resemble those which take place after the inoculation or absorption of an animal poison," are facts which have been still more recently pointed out by his able colleague, Dr. Roots.†

Armed with these authorities, we now turn back a few pages, to a chapter where the author has considered "the connexion of puerperal fever with erysipelas," (p. 142:) he commences as follows: "That puerperal fever has a decidedly inflammatory character, whatever be its cause, is now undisputed; and that phlebitis frequently attends it, is also undoubted." With the latter part of the sentence we of course agree; but we are surprised that the author should come to the conclusion "that puerperal fever has a decidedly inflammatory character," when, in the previous part of his work, he has so ably and so judiciously contradicted this too prevailing but (we conceive) erroneous opinion, and has shown so satisfactorily not only that puerperal fever is not necessarily accompanied by inflammation, but that it may exist entirely distinct from and independent of it. We refer our readers to the quotations we have made at p. 34 and 63 of the author's work, and particularly to the arguments which he adduces against Dr. Lee's opinion as to the inflammatory nature of puerperal fever, (p. 89-90,) where he not less distinctly than correctly shows that the symptoms of puerperal fever may run their fatal course, "and yet not a vestige of inflammation is to be detected in any of those tissues." He seems, as we have before observed, to have lost sight of the main object for which he has been contending, and to be carried out of his right course by the conflicting currents which surround him.

The subject of puerperal fever is confessedly one of no slight difficulty, and we candidly own that there is none the discussion of which we have always approached with more reluctance: still, however, we must not allow the author, after having advanced so far and so well, to fail at last, without reminding him of what he has already achieved in the preceding parts of his work.

That puerperal fever does frequently bear a strong analogy to certain forms of erysipelas, cannot be doubted: we have already pointed out the similarity of their origin. That a similar identity exists between the worst forms of uterine phlebitis and these species of erysipelas, we have also endeavoured to show. This brings us to the author's observations

* St. Thomas's Hospital Reports, No. III. p. 327. † Op. citat. p. 358-9.

on the contagiousness of puerperal fever: these are short and judicious, and tend to prove that puerperal fever may be distinctly communicated by contagion: he resumes his former excellent train of argument, and says, "the facts bearing on the contagious nature of puerperal fever are numerous and forcible. Those writers who have espoused the opinion that this fever is simply the effect of local inflammation are, of course, opposed to the notion of its being contagious; but, if the facts recorded cannot be invalidated, their opposition must prove futile." (P. 149.) The diseases in the same class to which it belongs are all capable of propagation by contagion, and most, if not all of them, by an epidemic constitution of the air. Experience has proved that puerperal fever may be communicated by either of these means; facts have moreover occurred within our own personal knowledge, of the disease being more or less communicable to those not in the puerperal state. In one case, a woman who washed the linen belonging to a patient who had sunk under a short but violent attack of puerperal fever, was seized with diffuse cellular inflammation of the hand, which terminated in extensive sloughing.

The importance of ventilation and dry air as a preventive means are shown by the experience of Dr. Collins and M. Caillard.

"The experience of M. Caillard, in the lying-in wards of the Hôtel Dieu, from January to May, 1831, seems fully to prove that changes of temperature and humidity have great influence over the disease; for, in consequence of his determination that his patients should enjoy a wholesome atmosphere, he made great changes in their hygienic treatment, the result of which was that, instead of a mortality of one in seven, it soon became only one in seventy-seven." (P. 197.)

From Dr. Collins we have largely quoted on this subject in our third Number.

The author informs us, (p. 201,) "It does not appear that the disinfecting and purifying vapour of chlorine and other acids has ever been tried in this disease. The fœtor in lying-in hospitals is at times almost intolerable, from which, arising (as it evidently does) from the decomposition of animal matter, the experiments of Magendie would lead us to expect deleterious effects. Chlorine would counteract the poison in this as in other instances." We will not quarrel with the author for calling chlorine an acid, but we must differ from him as to the fact of chlorine, &c. not being tried in this disease, and we refer him to Dr. Collins's work. We can also assure him that chlorine has been, and is constantly employed at similar institutions in the metropolis.

We fully agree with him in the importance which he attaches to preventing any offensive accumulation of the lochial and vaginal discharges, by the proper employment of injections, &c. But we have already made some observations on this subject in our review of Dr. Collins's work, and need not therefore repeat them. We are glad to find that injections of warm water into the vagina are advocated by the author.

In speaking of bleeding, local as well as general, the author very properly deprecates the French practice of employing such immense numbers of leeches, and justly observes, that "the debility produced by the hemorrhage from one or two hundred leech-bites must be often so great as to preclude recovery." That leeches "mây be advantageously employed under certain circumstances," we fully agree with him. Mr. Moore terminates his observations on bleeding with the following sound remark:

"When the cases of puerperal fever are comparatively few, although still epidemic, or when they are declining in frequency,—that is, when the cause of the disease is less active,—they are such as venesection generally relieves, and often completely and quickly cures. The symptoms in such cases are great increase of heat, acute local pain, distinct hard pulse. Bleeding, however, is of little avail when the muscles become flaccid, the skin clammy and relaxed, the pulse small and frequent. Pyrexial heat is rarely developed under the latter circumstances, since the vital energy is suppressed." (P. 217.)

The value of purgatives is admitted, but at the same time duly qualified by the following remark:

"That early purgation is indeed an essential auxiliary in the treatment, is amply testified by those who have been most successful; but that it alone should be capable of arresting the progress of so fierce and rapid a disease, is scarcely credible. We are ready to conclude, that Richter and others cured irritation of the bowels with purgation, but discovered the phænomena of puerperal fever only with the scalpel." (P. 223.)

Mr. Moore speaks highly, and yet judiciously, of the value of mercury, and does not appear to look forward to inducing salivation as the effect necessary to be produced before relief can be obtained.

"When guarded by opium or Dover's powder, and administered in small doses at suitable intervals, it offers the best auxiliary to allay the general irritability which accompanies this fever. Unless, however, the inflammatory tendency be previously subdued, its utility is at best but questionable, unless when so exhibited as to act briskly on the bowels. In this way large doses, under skilful management, have certainly been prescribed with great advantage." . . . "Yet, though mercury brings the system completely under its specific influence, it does not possess any power to check the course of the disease." (P. 225-6.)

He quotes Dr. Dewees's excellent remarks *against* producing the specific action of mercury upon the salivary glands, with which we fully agree. In the worst forms of puerperal fever, we must confess that we have never seen much benefit arise from the use of mercury, except where it had been given in one or two large doses at the beginning, so as to produce a general increase of all the excretions.

With regard to alkalies, the author mentions their having been found useful in the practice of Mascagni, Guniot, Allen, and especially M. Recamier, who gave the carbonate of potass. We have before mentioned that we have found advantage from them in puerperal epidemics, when everything else had seemed to be without effect; but we must have further confirmation of this fact before any distinct inference can be drawn.

The advantage of fomentations is shown, and a short summary given of the different opinions respecting the employment of turpentine.

With this we close our review of Mr. Moore's work. If we might presume to advise the alteration of those passages to which we have objected, (and in which, upon impartial consideration, we are sure he will agree with us,) we feel confident that the second edition, which the work well deserves, will hold a very high rank among treatises upon this subject: as it is, it is decidedly a work of great merit, and is an honorable proof of its author's talents and acquirements. The getting-up of the book is good, but the proper names are frequently misprinted.

